# Association between fingertip-measured advanced glycation end products and cardiovascular events in outpatients with cardiovascular disease

**DOI:** 10.1186/s12933-023-01953-x

**Published:** 2023-08-17

**Authors:** Tomoya Hirai, Kazuhiro Fujiyoshi, Satoru Yamada, Takuya Matsumoto, Junko Kikuchi, Kohki Ishida, Miwa Ishida, Kyo Shigeta, Taiki Tojo

**Affiliations:** 1 Division of Rehabilitation, Department of Clinical Practice and Support, Hiroshima University Hospital, Hiroshima, Japan; 2 Department of Cardiac Rehabilitation, Kitasato University Kitasato Institute Hospital, Minato-Ku, Japan; 3 Department of Cardiovascular Medicine, Kitasato University School of Medicine, 1-15-1 Kitasato, Minami-ku, Sagamihara, 252-0373 Japan; 4 Diabetes Center, Kitasato University Kitasato Institute Hospital, Minato-ku, Japan; 5 Department of Cardiovascular Medicine, Kitasato University Kitasato Institute Hospital, Minato-ku, Japan

**Keywords:** Advanced glycation end products, Noninvasive examination, Fingertip, Major adverse cardiac and cerebrovascular events, Secondary prevention, Outpatient cardiac rehabilitation

## Abstract

**Background:**

The accumulation of advanced glycation end products (AGEs) is associated with cardiovascular events in patients with cardiovascular disease (CVD). However, the relationship between the AGEs measured by an AGEs sensor noninvasively at the fingertip and prognosis in patients with CVD remains unclear. Therefore, this study aimed to determine the relationship between AGEs score and prognosis among patients with CVD.

**Methods:**

A total of 191 outpatients with CVD were included. AGEs score were measured using an AGEs sensor and the patients were classified into groups by the median value of AGEs score. The incidence of major adverse cardiovascular and cerebrovascular events (MACCE) at 30 months was compared between high- and low-AGEs score groups. In addition, receiver operating characteristic (ROC) curve analysis was used to calculate cutoff value for the AGEs score, which discriminates the occurrence of MACCE. Cox regression analysis was performed to identify the factors associated with the presence of MACCE. MACCE included cardiac death, myocardial infarction, percutaneous coronary intervention, heart failure, and stroke.

**Results:**

AGEs score was normally distributed, with a median value of 0.51. No significant intergroup differences were found in laboratory findings, physical functions, or medications. The high-AGEs score group had a significantly higher incidence of MACCE than the low-AGEs score group (27.1 vs. 10.5%, *P* = 0.007). A high-AGEs score was a risk factor for MACCE (hazard ratio, 2.638; 95% confidence interval, 1.271–5.471; *P* = 0.009). After the adjustment for confounders other than 6-min walking distance, the AGEs score remained a factor associated with the occurrence of MACCE. The best cutoff AGEs score for the detection of MACCE was 0.51 (area under the curve, 0.642; *P* = 0.008; sensitivity, 72.2%; specificity, 54.8%).

**Conclusions:**

AGEs score measured at the fingertip in patients with CVD is associated with MACCE. AGEs score, which can be measured noninvasively and easily, may be useful as an assessment for the secondary prevention of CVD in patients with CVD.

**Supplementary Information:**

The online version contains supplementary material available at 10.1186/s12933-023-01953-x.

## Background

The incidence of cardiovascular disease (CVD) increases with age worldwide [1]. In aging countries, the number of patients with CVD is significantly increasing [2]. Despite significant advances in treatments and outcomes, the rehospitalization rate for CVD remains high [3]. Furthermore, patients readmitted with CVD recurrence have higher all-cause and cardiovascular mortality rates [3]. Given the increasing number of older patients with CVD, it is important to identify factors that predict recurrent CVD for secondary prevention. Previous studies clarified that physical functions such as muscle strength [4, 5] and exercise tolerance (e.g., 6-min walk distance [6MWD]) [6–8] predict the prognosis of patients with CVD. Cardiac rehabilitation including physical evaluations is useful for the secondary prevention of CVD [9]. However, performing these evaluations in older patients with CVD may be difficult. Intima-media thickness (IMT) and pulse-wave velocity (PWV) are also available [10, 11]. Nevertheless, maintaining intra- and interrater reliability is difficult.

Serum advanced glycation end products (AGEs) are excellent predictors of CVD events [12–14]. However, these tests are invasive. AGEs can now be measured noninvasively and conveniently as AGEs score by an instrument called an AGEs sensor [15]. A previous study reported that the AGEs score is associated with lower limb muscle strength and 6MWD in patients with CVD [16]. Because of its noninvasive and simple measurement, the AGEs sensor may be applicable to the evaluation of physical function in outpatients of cardiac rehabilitation. Furthermore, it may be a useful prognostic indicator in patients with CVD. However, it is unclear whether the AGEs score in patients with CVD is a predictor of CVD events. In patients with CVD, determining the association between the AGEs score, a noninvasive and simple measure of AGEs, and CVD events may provide useful information for the prevention of recurrent CVD.

Therefore, this study aimed to determine the association between the AGEs score measured at the fingertip and CVD events in patients with CVD.

## Methods

### Study population

This single-center retrospective observational study was conducted between August 2020 and January 2023 at the Cardiac Rehabilitation Center of Kitasato University Kitasato Institute Hospital. We enrolled 204 patients with CVD who underwent cardiac rehabilitation. CVD diagnoses included ischemic heart disease (myocardial infarction [MI], angina pectoris, and vasospastic angina), heart failure (HF), valvular heart disease, and atrial fibrillation. After excluding patients for whom AGEs score was not measured at the start of cardiac rehabilitation (n = 2) and those lost to follow-up (n = 11), 191 patients were ultimately included in our study (Additional file 1: Fig. S1). In this study, the sample size was calculated by a previous study dealing with the association between AGEs and clinical events in heart failure patients [17]. The Kitasato Institute Hospital Research Ethics Committee approved the study protocol (clinical trial registration no. 21028).

### Assessment of the AGEs score

To estimate the AGEs score, skin autofluorescence (sAF) levels were measured at the start of cardiac rehabilitation using an AGEs sensor (AIR WATER BIODESIGN; Kobe, Japan). AGEs emit fluorescence when irradiated with a specific excitation light. The AGEs sensor irradiates the fingertip with excitation light, acquires percutaneous fluorescence from them, and measures skin autofluorescence. The sAF levels were measured at the middle finger of the left hand where the least amount of skin melanin was present. We took the sAF measurements twice before cardiac rehabilitation and used the mean values in the analysis. The measured AGEs values are expressed as AGEs score in arbitrary units with an upper limit of 10.0 and a lower limit of 0.0. According to a recent manufacturer survey, 0.5 is an arbitrary unit that corresponds approximately to the average score of healthy Japanese patients aged 50 years. A previous study demonstrated that the AGEs sensor is useful for the noninvasive assessment of glycation stress [18]. The AGEs sensor used in this study reflects methylglyoxal, a major precursor of AGEs formation. It is technical difficult in measuring AGEs with the AGEs sensor [15]. Furthermore, there is an uncertainty in AGEs measurement by the AGEs sensor. A previous study has reported a coefficient of variation of 6.7 ± 7.3% and an intra-class correlation coefficient (Cronbach’s alpha) of 0.938 when AGEs are measured repeatedly three times [19].

### Assessment of physical functions

We evaluated physical functions including handgrip strength, knee extension strength, and 6MWD. Handgrip strength was measured using a dynamometer (TKK 5401; Takei, Tokyo, Japan). In the sitting position with the elbow joint at 90 degrees of flexion, two 3-s maximum isometric contractions were measured on each hand. The width of the dynamometer handle was adjusted to match each participant’s hand size. The maximum weight (kg) was used for the analysis. Knee extension strength was measured using a handheld dynamometer (μ-Tas; ANIMA, Tokyo, Japan). Maximum isometric contractions of the quadriceps were measured twice in both lower extremities for 5 s with the participant in a sitting position with the hip and knee joints at 90 degrees of flexion. The measured maximum weight (kg) divided by body weight (%BW) was used in the analysis. The 6MWD was measured according to American Thoracic Society guidelines [20]. A chair was placed on a flat walking corridor in front of a straight 10-m line, and the patients were instructed to walk at their own pace. Walking distance (m) was used in the analysis.

### Cardiac rehabilitation

Cardiac rehabilitation was defined as participation in at least one cardiac rehabilitation session within 1 month of discharge. The frequency of outpatient cardiac rehabilitation visits was recorded for each patient. Exercise therapy consisted mainly of preparatory exercises such as stretching and moderate-intensity aerobic exercise based on the Japanese Circulation Society guidelines [9]. Aerobic exercise was performed at the anaerobic metabolic threshold calculated by the cardiopulmonary exercise testing or heart rate calculated by Karvonen’s formula (k = 0.4–0.6) or at an intensity of 12–13 on the Borg scale. Balance training was added depending on the patient’s physical function. Resistance training of the upper and lower extremities was performed after stabilization. Comprehensive instructions on disease management were also provided to patients and their families as needed.

### Definitions

HF with reduced ejection fraction (HFpEF) was defined as a left ventricular ejection fraction (LVEF) < 40%, HF with a midrange ejection fraction (HFmrEF) as an LVEF of 40–49%, and HF with a preserved ejection fraction (HFpEF) as an LVEF > 50%. Hypertension was defined as an arterial blood pressure of ≥ 140/90 mmHg or the use of antihypertensive medication. Dyslipidemia was defined as low-density lipoprotein cholesterol level ≥ 140 mg/dL or the use of antidyslipidemic medication. Diabetes mellitus was defined as symptoms of diabetes plus a random plasma glucose concentration ≥ 200 mg/dL, fasting plasma glucose concentration ≥ 126 mg/dL, or the use of antihyperglycemic medication. Chronic kidney disease was defined as an estimated glomerular filtration rate < 60 mL/min/1.73 m^2^.

### Main outcome

The primary outcome of this study was major adverse cardiac and cerebrovascular events (MACCE), which included cardiac death, MI, percutaneous coronary intervention (PCI) attempts, HF, and stroke. The occurrence of MACCE was investigated using the medical records of Kitasato University Kitasato Institute Hospital. This study population was recruited at a cardiac rehabilitation center. Patients with ischemic heart disease and chronic heart failure were mainly included. These outcomes were applied MACCE including rehospitalization for heart failure and revascularization for ischemic heart disease in addition to typical 3-point MACE according to a previous study [17]. Follow-up surveys were conducted for up to 30 months after the initiation of cardiac rehabilitation.

### Statistical analysis

Normally distributed continuous variables are expressed as mean ± standard deviation, whereas non-normally distributed values are expressed as median with interquartile range. We compared the basic characteristics of patients with versus without MACCE. Continuous variables were analyzed using unpaired t-tests. Categorical variables are reported as number (%) and were analyzed using the chi-squared test. Kaplan–Meier curves were used to analyze the association between AGEs score and MACCE. In the analysis, the baseline AGEs score was divided into two groups according to the median value. Differences in the rate of MACCE according to AGEs score at baseline were analyzed using the log-rank test. Receiver operating characteristic (ROC) curves were constructed to assess the predicted occurrence of MACCE and to determine the best cutoff of the AGEs score for the detection of MACCE. A multivariate regression analysis was performed to identify the factors associated with the presence of MACCE among the variables with values of *P* < 0.050 (body mass index (BMI), heart failure, ischemic heart disease, brain natriuretic peptide (BNP), handgrip strength, knee extension strength, 6MWD, stable angina pectoris, diuretic) on the univariate Cox regression analysis. In the multivariate analysis, AGEs score suggested a stronger influence on the occurrence of MACCE by adjusting the factors exhibiting significant differences in the univariate analysis. Statistical significance was defined as *P* < 0.050. SPSS 27 version (IBM Corporation, Armonk, NY, USA) was used to perform all statistical analyses. In this study, the AGEs score were normally distributed (Additional file 1: Fig. S2). We defined values above and below the median as the high-AGEs and low-AGEs groups, respectively. Thus, the median AGEs score (0.51) was classified into two groups (high- and low-AGEs) and subsequently analyzed.

## Results

### Baseline characteristics

There were 96 (50.3%) and 95 (49.7%) patients in the high- and low-AGEs groups, respectively. The mean AGEs score of the high- and low-AGEs groups were 0.59 ± 0.06 and 0.43 ± 0.06, respectively. Table 1 and Additional file 1: Table S1 compare the clinical characteristics of the high- and low-AGEs groups. No significant intergroup differences were noted in laboratory findings such as HbA1c (6.3 ± 1.0 vs. 6.2 ± 0.8%, *P* = 0.430) or physical functions such as handgrip strength (26.0 ± 9.7 vs. 27.0 ± 8.9 kg, *P* = 0.468), knee extension strength (42.9 ± 15.2 vs. 44.0 ± 13.6%BW, *P* = 0.580), or 6MWD (397 ± 148 vs. 429 ± 130 m, *P* = 0.123).

**Table 1:** Comparison of baseline characteristics of patents with high AGEs and low AGEs score

	High-AGEs group (n = 96)	Low-AGEs group (n = 95)	*P* value
Age, year	73.1 ± 13.5	73.2 ± 11.9	0.945
Male, n (%)	62 (65)	65 (68)	0.574
Body mass index, kg/m^2^	23.9 ± 4.2	23.5 ± 4.4	0.492
Cardiovascular diseases			
Heart failure, n (%)	54 (56)	52 (55)	0.833
Ischemic heart disease, n (%)	43 (45)	43 (45)	0.948
Valvular disease, n (%)	13 (14)	9 (9)	0.379
Cardiomyopathy, n (%)	11 (11)	12 (13)	0.803
Atrial fibrillation, n (%)	27 (28)	25 (26)	0.799
Cardiovascular disease risk factors			
Hypertension, n (%)	55 (57)	46 (48)	0.219
Hyperlipidemia, n (%)	36 (38)	37 (39)	0.837
Diabetes mellitus, n (%)	32 (33)	26 (26)	0.370
Past smoker, n (%)	27 (28)	30 (32)	0.529
Chronic kidney disease, n (%)	56 (58)	60 (63)	0.495
Laboratory findings			
LDL-cholesterol, mg/dL	88.1 ± 31.2	86.8 ± 30.1	0.781
Blood glucose, mg/dL	123.2 ± 37.0	114.5 ± 30.0	0.108
HbA1c, %	6.3 ± 1.0	6.2 ± 0.8	0.430
Creatinine, mg/dL	1.16 ± 0.88	1.15 ± 0.87	0.935
eGFR, mL/min/1.73 m^2^	55.6 ± 22.8	60.0 ± 19.5	0.463
BNP, pg/dL	231 ± 408	233 ± 248	0.969
LVEF, %	57 ± 13	54 ± 13	0.217
AGEs score	0.59 ± 0.06	0.43 ± 0.06	< 0.001*
Physical functions			
Handgrip strength, kg	26.0 ± 9.7	27.0 ± 8.9	0.468
Knee extension strength, %BW	42.9 ± 15.2	44.0 ± 13.6	0.580
6MWD, m	397 ± 148	429 ± 130	0.123

The date are means ± standard deviation or number (%), high-AGEs group vs. low-AGEs group

*AGEs* advanced glycation end products, *BNP* brain natriuretic peptide, *eGFR* estimated glomerular filtration rate, *HbA1c* hemoglobin-A1c, *LDL* low-density lipoprotein, *LVEF* left ventricular ejection fraction, *6MWD* 6-min walk distance

^*^P < 0.050

### Clinical outcomes (MACCE) at 30 months

**Fig. 1:**
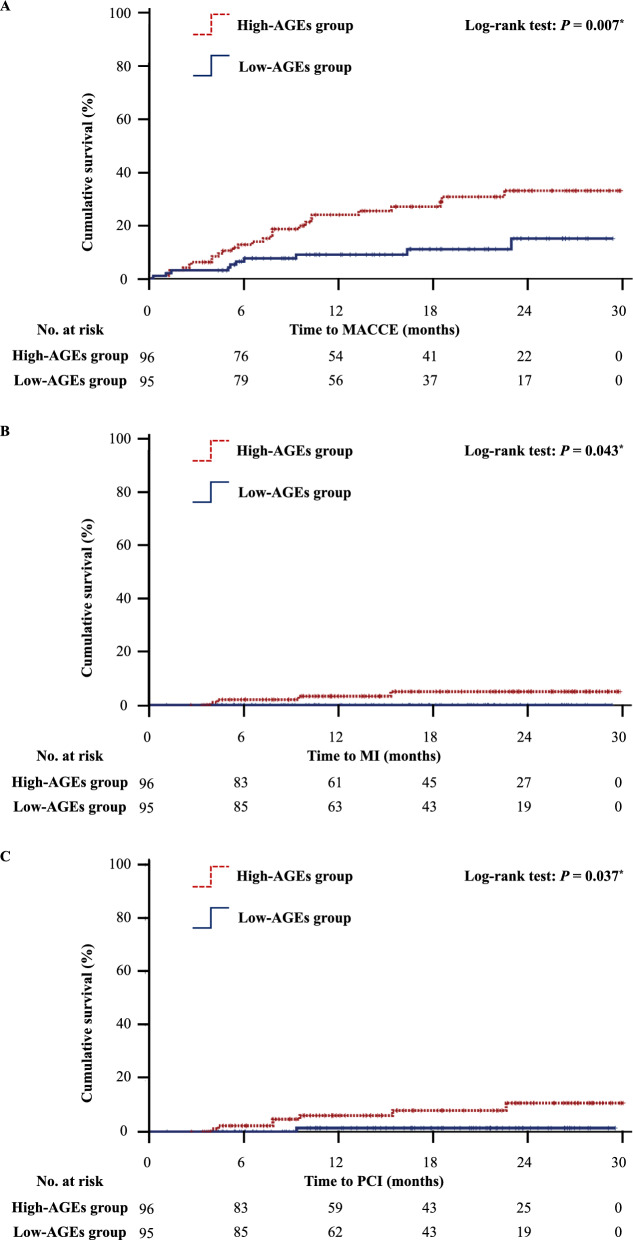
Kaplan–Meier curves show the comparison of the incidence of cardiovascular events in the high- and low-advanced glycation end products (AGEs) groups. The incidence of major adverse cardiac and cerebrovascular events (**A** 27.1 vs. 10.5%, *P* = 0.007), myocardial infarction (**B** 4.2 vs. 0.0%, *P* = 0.043), and percutaneous coronary intervention attempts (**C** 7.3 vs. 1.1%, *P* = 0.037) were significantly higher in the high-AGEs group than in the low-AGEs group

The mean follow-up period was 15.1 ± 8.4 months, and 36 patients (18.8%) had a MACCE. The high-AGEs group had a significantly higher incidence of MACCE than the low-AGEs group (27.1 vs. 10.5%, *P* = 0.007). In terms of MACCE, the high-AGEs group had a significantly higher incidence of MI (4.2 vs. 0.0%, *P* = 0.043) and PCI attempts (7.3 vs. 1.1%, *P* = 0.037) than the low-AGEs group, and no significant difference was found in other events (Table 2). The incidence of MACCE at 30 months is shown in Fig. 1 and Additional file 1: Fig. S3. The incidence of MACCE was significantly higher in the high- versus low-AGEs group (Fig. 1A; 27.1 vs. 10.5%, *P* = 0.007). Furthermore, the incidence of MI (Fig. 1B; 4.2 vs. 0.0%, *P* = 0.043) and PCI attempts (Fig. 1C; 7.3 vs. 1.1%, *P* = 0.037) were significantly higher in the high-versus low-AGEs group. There was no significant difference in all-cause death, cardiac death, heart failure, and stroke rates between the high- and low-AGEs groups (Additional file 1: Fig. S3).

**Table 2:** Clinical outcomes at 30 months

	Overall (n = 191)	High-AGEs group (n = 96)	Low-AGEs group (n = 95)	*P* value
All cause death, n (%)	13 (7)	9 (9)	4 (4)	0.156
MACCE, n (%)	36 (19)	26 (27)	10 (11)	0.003*
Cardiac death, n (%)	7 (4)	5 (5)	2 (2)	0.254
Myocardial infarction, n (%)	4 (2)	4 (4)	0 (0)	0.044*
PCI, n (%)	8 (4)	7 (4)	1 (1)	0.031*
Heart failure, n (%)	25 (13)	16 (17)	9 (9)	0.141
Stroke, n (%)	2 (1)	2 (2)	0 (0)	0.157

High-AGEs group vs. low-AGEs group

*AGEs* advanced glycation end products, *MACCE* major adverse cardiac and cerebrovascular events, *PCI* percutaneous coronary intervention

*P < 0.050

### Association between the AGEs score and MACCE

The univariate analysis showed that BMI (hazard ratio [HR], 0.900; 95% confidence interval [CI] 0.809–0.983; *P* = 0.019), HF (HR, 2.595; 95% CI 1.220–5.518; *P* = 0.013), ischemic heart disease (HR, 0.415; 95% CI 0.200–0.860; *P* = 0.018), BNP (HR, 1.001; 95% CI 1.000–1.001; *P* < 0.001), AGEs score (HR, 1.044; 95% CI 1.007–1.081; *P* = 0.018), handgrip strength (HR, 0.956; 95% CI 0.919–0.993; *P* = 0.022), knee extension strength (HR, 0.975; 95% CI 0.951–1.000; *P* = 0.046), 6MWD (HR, 0.995; 95% CI 0.992–0.998; *P* < 0.001), stable angina pectoris (HR, 0.383; 95% CI 0.168–0.875; *P* = 0.023), and diuretic use (HR, 3.150; 95% CI 1.575–6.301; *P* = 0.001) were significantly associated with the incidence of MACCE (Table 3, Additional file 1: Table S2). After the adjustment for confounders other than 6MWD, the AGEs score remained a factor associated with the occurrence of MACCE (Table 4, Additional file 1: Table S3).

**Table 3:** Univariate analysis of factors associated with MACCE

Variable	HR [95% CI]	*P* value
Age, per year	1.029 [0.999–1.059]	0.059
Male	0.639 [0.329–1.240]	0.186
Body mass index, per kg/m^2^	0.900 [0.824–0.983]	0.019*
Cardiovascular diseases		
Heart failure	2.595 [1.220–5.518]	0.013*
Ischemic heart disease	0.415 [0.200–0.860]	0.018*
Valvular disease	1.576 [0.655–3.790]	0.310
Cardiomyopathy	1.141 [0.444–2.935]	0.784
Atrial fibrillation	1.955 [1.000–3.823]	0.050
Cardiovascular disease risk factors		
Hypertension	1.107 [0.573–2.139]	0.762
Hyperlipidemia	0.591 [0.285–1.226]	0.158
Diabetes mellitus	0.767 [0.361–1.632]	0.491
Past smoker	0.802 [0.387–1.664]	0.554
Chronic kidney disease	1.349 [0.674–2.760]	0.397
Laboratory findings		
LDL-cholesterol, mg/dL	1.001 [0.990–1.013]	0.848
Blood glucose, per mg/dL	0.997 [0.985–1.009]	0.613
HbA1c, per %	0.598 [0.348–1.027]	0.062
Creatinine, per mg/dL	1.099 [0.780–1.549]	0.588
eGFR, per mL/min/1.73 m^2^	0.986 [0.970–1.003]	0.104
BNP, per pg/dL	1.001 [1.000–1.001]	< 0.001*
LVEF, per %	0.982 [0.961–1.005]	0.120
AGEs score, per 0.01	1.044 [1.007–1.081]	0.018*
Physical functions		
Handgrip strength, per kg	0.956 [0.919–0.993]	0.022*
Knee extension strength, per %BW	0.975 [0.951–1.000]	0.046*
6MWD, per m	0.995 [0.992–0.998]	< 0.001*

*AGEs* advanced glycation end products, *BNP* brain natriuretic peptide, *CI* confidence interval, *eGFR* estimated glomerular filtration rate, *HbA1c* hemoglobin-A1c, *HR* hazard ratio, *LDL* low-density lipoprotein, *LVEF* left ventricular ejection fraction, *MACCE* major adverse cardiac and cerebrovascular events, *6MWD* 6-min walk distance

*P < 0.050

**Table 4:** Multivariate analysis of factors associated with MACCE

	HR [95% CI]	*P* value	HR [95% CI]	*P* value	HR [95% CI]	*P* value	HR [95% CI]	*P* value
AGEs score	1.055 [1.016–1.095]	0.005*	1.043 [1.007–1.080]	0.019*	1.042 [1.007–1.079]	0.019*	1.031 [1.001–1.068	0.048*
Body mass index	0.882 [0.805–0.966]	0.007*						
Heart failure			2.576 [1.211–5.478]	0.014*				
Ischemic heart disease					0.418 [0.201–0.867]	0.019*		
BNP							1.001 [1.000–1.001]	0.001*

*AGEs* advanced glycation end products, *BNP* brain natriuretic peptide, *CI* confidence interval, *HR* hazard ratio, *MACCE* major adverse cardiac and cerebrovascular events

*P < 0.050

### Calculation of the AGEs score and 6MWD cutoff values for predicting MACCE

**Fig. 2:**
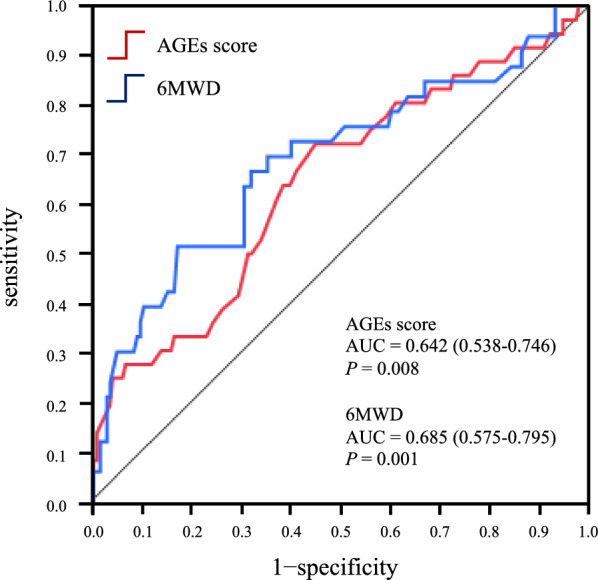
Receiver-operating characteristic (ROC) curves analysis of the advanced glycation end products (AGEs) score and 6-min walk distance (6MWD) to compare their diagnostic performance in detecting major adverse cardiac and cerebrovascular events (MACCE). The cut-off values for the AGEs score and 6MWD discriminating the occurrence of MACCE were 0.51 and 378 m, respectively. The accuracy of the AGEs score and 6MWD in identifying MACCE was 72.2% and 54.8% for sensitivity and 67.8% and 66.7% for specificity, respectively

The areas under the ROC curve for the detection of MACCE assessed using AGEs score and 6MWD were 0.642 (95% CI 0.538–0.746; *P* = 0.008) and 0.685 (95% CI 0.575–0.795; *P* = 0.001), respectively (Fig. 2). The best cutoff values for AGEs score and 6MWD were 0.51 (sensitivity, 72.2%; specificity, 54.8%) and 378 m (sensitivity, 67.8%; specificity, 66.7%), respectively.

## Discussion

The main findings of this study were as follows: (1) in patients with CVD, an association was identified between AGEs measured at the fingertip and MACCE; (2) in patients with CVD, the AGEs score was as predictive of cardiovascular events as 6MWD; and (3) the AGEs score that predicts MACCE in patients with CVD was 0.51.

### Association between the AGEs score and MACCE

This study showed a correlation between AGEs measured at the fingertip and MACCE in patients with CVD. Previous studies reported a connection between serum AGEs [11–13] and forearm-measured AGEs [17, 21–24] and MACCE including cardiovascular death, MI, and stroke. Furthermore, several meta-analyses [23, 24] indicated that AGEs accumulation affects MACCE. However, the relationship between AGEs measured at the fingertip and MACCE has not been fully elucidated. This study demonstrated that the AGEs score, which can be easily and noninvasively measured at the fingertip, is associated with MACCE. These findings support those of previous studies and suggest that the AGEs score is a useful tool for managing in patients with CVD, as it can be measured quickly and easily. Laboratory findings and physical function tests such as the 6 MWD, which are associated with the occurrence of MACCE in patients with CVD, are invasive and cannot be applied to older patients with difficulty walking. However, the AGEs score can be measured noninvasively and used to evaluate MACCE in patients who have difficulty performing physical function tests.

### Mechanism of association between the AGEs score and MACCE

AGEs inhibit nitric oxide activity in the vascular endothelium, produce reactive oxygen species, and contribute to the progression of atherosclerosis [25]. Additionally, a previous study reported that AGEs accumulation is associated with indicators of atherosclerosis, such as IMT and PWV [26]. IMT and PWV are indicators of arterial stiffness and are related to the occurrence of MACCE [9, 10]. AGEs induce intracellular oxidative stress via receptors on vascular endothelial cells. Oxidative stress induces an inflammatory response and thrombotic tendencies by inactivating nitric oxide, which contributes to the development of atherosclerosis. Thus, the accumulation of AGEs is associated with the occurrence of MACCE.

### Potential of the AGEs score as a predictor of MACCE

AGEs are essential indicators for disease management in patients with CVD. Previous studies reported an association between the AGEs score measured at the fingertip, knee extension strength, and 6MWD [15] as well as between the AGEs score measured at the forearm and maximal oxygen uptake [27]. AGEs accumulation decreases muscle function by increasing muscle hardness and decreasing viscoelasticity. Additionally, the crosslinking of myocardial collagen with the AGEs score may contribute to increased myocardial stiffness and diastolic dysfunction [28, 29], which can result in limited cardiac reserve during exercise and limited exercise tolerance. Muscle strength [4, 5] and exercise tolerance [6–8] are strongly associated with MACCE in patients with CVD. Based on our results, assessing the AGEs score in patients with CVD is essential for risk assessment and management.

### Potential of cardiac rehabilitation for measuring the AGEs score

Drugs, diet, and physical therapies reportedly reduce AGEs levels. AGEs inhibitors, angiotensin II receptor blockers, and angiotensin-converting enzyme inhibitors reduce AGEs levels [30]. This mechanism mainly involves reducing the production of reactive carbonyl and dicarbonyl compounds, which are AGEs precursors, through oxidative metabolism [31]. AGEs also reportedly accumulate from dietary sources [32], and dietary therapy (foods low in AGEs) improves AGEs accumulation, insulin resistance, and inflammatory responses, making dietary therapy useful [33]. A previous study reported that community-dwelling older adults with higher AGEs score collected at the forearm had lower physical activity levels [34]. In addition, regular exercise reportedly reduces peripheral resistance to insulin and delays or reduces AGEs accumulation [35]. Comprehensive cardiac rehabilitation, including exercise and diet therapy, is expected to effectively prevent all cardiac diseases [36]. Cardiac rehabilitation in patients with CVD improves exercise tolerance and muscle strength [37, 38]. However, few studies have examined the effects of exercise interventions on AGEs. Cardiac rehabilitation in particular may contribute significantly to the AGEs score reduction because it is a comprehensive program that includes exercise therapy as well as drug and diet therapies. However, the effects of cardiac rehabilitation on AGEs remain unclear. Therefore, further longitudinal verification is required.

### Study limitations

The present study has several limitations. First, generalizations cannot be made because it was validated at a single institution. Second, the AGEs score was measured only at baseline, and a causal relationship was unclear. Third, although the participants in this study underwent cardiac rehabilitation, the frequency and content of the programs were not standardized among patients, which may have affected the results. Fourth, because this study included several diseases such as ischemic heart disease and HF, the cutoff values may have differed for each disease. Fifth, this study is an exploratory study and is limited by sample size.

## Conclusions

The AGEs score in patients with CVD was associated with the occurrence of secondary CVD events. The AGEs score measured at the fingertip may be a useful indicator of MACCE risk.

### Supplementary Information

**Additional file 1: Figure S1.** Study flowchart. AGEs; advanced glycation end products. **Figure S2.** Normality test of advanced glycation end products (AGEs) score by Kolmogorov–Smirnov test. The average AGEs score was 0.51, and the median value was 0.51, indicating a normal distribution (*P* = 0.200). **Figure S3.** Kaplan–Meier curves show the comparison of the incidence of cardiovascular events in the high- and low-advanced glycation end products (AGEs) groups. The incidence of all-cause death (A, 9.4 vs. 4.2%, P = 0.296), cardiac death (B, 5.2 vs. 2.1%, P = 0.317), heart failure (C, 16.7 vs. 9.5%, P = 0.185), stroke (D, 1.0 vs. 0.0%, P = 0.156) were not significantly different between the high- and low-AGEs groups. **Table S1.** Baseline clinical characteristics. **Table S2.** Univariate analysis of factors associated with MACCE. **Table S3.** Multivariate analysis of factors associated with MACCE in physical functions. **Table S4.** Multivariate analysis of factors associated with MACCE.

## Data Availability

The datasets used and/or analyzed during the current study are available from the corresponding author on reasonable request.

## Abbreviations

CVD: Cardiovascular disease
6MWD: 6-Min walk distance
IMT: Intima-media thickness
PWV: Pulse-wave velocity
AGEs: Advanced glycation end products
MI: Myocardial infarction
HF: Heart failure
sAF: Skin autofluorescence
HFrEF: HF with reduced ejection fraction
LVEF: Left ventricular ejection fraction
HFmrEF: HF with midrange ejection fraction
HFpEF: HF with ejection fraction
MACCE: Major adverse cardiac and cerebrovascular events
PCI: Percutaneous coronary intervention
ROC: Receiver operating characteristic
BMI: Body Mass Index
BNP: Brain natriuretic peptide
HR: Hazard ratio
CI: Confidence interval
