# Supplementary material for: Association between fingertip-measured advanced glycation end products and cardiovascular events in outpatients with cardiovascular disease
Source: Cardiovasc Diabetol. 2023 Aug 17;22:213. doi: 10.1186/s12933-023-01953-x (PMC10436644; doi:10.1186/s12933-023-01953-x)
Supplement: Supplementary file 1 — Additional file 1: Figure S1. Study flowchart. AGEs; advanced glycation end products. Figure S2. Normality test of advanced glycation end products (AGEs) score by Kolmogorov–Smirnov test. The average AGEs score was 0.51, and the median value was 0.51, indicating a normal distribution (P = 0.200). Figure S3. Kaplan–Meier curves show the comparison of the incidence of cardiovascular events in the high- and low-advanced glycation end products (AGEs) groups. The incidence of all-cause death (A, 9.4 vs. 4.2%, P = 0.296), cardiac death (B, 5.2 vs. 2.1%, P = 0.317), heart failure (C, 16.7 vs. 9.5%, P = 0.185), stroke (D, 1.0 vs. 0.0%, P = 0.156) were not significantly different between the high- and low-AGEs groups. Table S1. Baseline clinical characteristics. Table S2. Univariate analysis of factors associated with MACCE. Table S3. Multivariate analysis of factors associated with MACCE in physical functions. Table S4. Multivariate analysis of factors associated with MACCE. [file 12933_2023_1953_MOESM1_ESM.docx]

**Association between Fingertip-Measured Advanced Glycation End Products and Cardiovascular Events in Outpatients with Cardiovascular Disease**

Tomoya Hirai1, 2, Kazuhiro Fujiyoshi3*, Satoru Yamada4, Takuya Matsumoto2, Junko Kikuchi2, Kohki Ishida5, Miwa Ishida5, Kyo Shigeta2, Taiki Tojo5

1 Department of Rehabilitation, Hiroshima University Hospital, Hiroshima, Japan

2 Department of Cardiac Rehabilitation, Kitasato University Kitasato Institute Hospital, Minato-ku, Japan

3 Department of Cardiovascular Medicine, Kitasato University School of Medicine, Sagamihara, Japan

4 Diabetes Center, Kitasato University Kitasato Institute Hospital, Minato-ku, Japan

5 Department of Cardiovascular Medicine, Kitasato University Kitasato Institute Hospital, Minato-ku, Japan

* Corresponding author:

Kazuhiro Fujiyoshi, MD, PhD

Department of Cardiovascular Medicine, Kitasato University School of Medicine

1-15-1 Kitasato, Minami-ku, Sagamihara, 252-0373, Japan.

E-mail: kazuhiro@med.kitasato-u.ac.jp

Phone: (81)-42-778-9696, Fax: (81)-42-778-9696

**Additional Material**

Table S1–S4

Figures S1–S3

Additional Figure legends

Figure S1. Study flowchart. AGEs; advanced glycation end products.

Figure S2. Normality test of advanced glycation end products (AGEs) score by Kolmogorov–Smirnov test. The average AGEs score was 0.51, and the median value was 0.51, indicating a normal distribution (*P* = 0.200)

Figure S3. Kaplan-Meier curves show the comparison of the incidence of cardiovascular events in the high- and low-advanced glycation end products (AGEs) groups. The incidence of all-cause death (Figure S3-A, 9.4 vs. 4.2%, P = 0.296), cardiac death (Figure S3-B, 5.2 vs. 2.1%, P = 0.317), heart failure (Figure S3-C, 16.7 vs. 9.5%, P = 0.185), stroke (Figure S3-D, 1.0 vs. 0.0%, P = 0.156) were not significantly different between the high- and low- AGEs groups.

| Table S1. baseline clinical characteristics | | | | | | | | |
| --- | --- | --- | --- | --- | --- | --- | --- | --- |
|  | High-AGEs group (n = 96) | | | Low-AGEs group (n = 95) | | | *P* Value | |
|  |  |  |  |  |  |  |  |  |
| Cardiac rehabilitation, times / month | 2.2 | ± | 1.5 | 2.6 | ± | 1.7 |  | 0.099 |
| **Cardiovascular diseases** |  |  |  |  |  |  |  |  |
| HFrEF, n (%) | 10 (10) | | | 17 (18) | | |  | 0.138 |
| HFmrEF, n (%) | 6 (6) | | | 7 (7) | | |  | 0.759 |
| HFpEF, n (%) | 38 (40) | | | 28 (29) | | |  | 0.142 |
| Stable angina pectoris, n (%) | 31 (32) | | | 35 (37) | | |  | 0.508 |
| Acute coronary syndrome, n (%) | 18 (19) | | | 17 (18) | | |  | 0.879 |
| **Medications** |  | | |  | | |  | 0.357 |
| ARB, n (%) | 51 (53) | | | 51 (54) | | |  | 0.938 |
| ACE, n (%) | 13 (14) | | | 19 (20) | | |  | 0.232 |
| β blocker, n (%) | 61 (64) | | | 57 (60) | | |  | 0.615 |
| Calcium blocker, n (%) | 31 (32) | | | 25 (26) | | |  | 0.364 |
| Diuretic, n (%) | 39 (41) | | | 38 (40) | | |  | 0.930 |
| DPP4i, n (%) | 11 (11) | | | 10 (11) | | |  | 0.837 |
| Insulin, n (%) | 1 (1) | | | 2 (2) | | |  | 0.554 |
| Metformin, n (%) | 7 (7) | | | 7 (7) | | |  | 0.984 |
| SGLT2i, n (%) | 20 (21) | | | 27 (28) | | |  | 0.223 |
| The date are means ± standard deviation or number (%), High-AGEs group vs. Low-AGEs group, ^*^; *P* < 0.050, ACE; angiotensin-converting enzyme inhibitor, AGEs; advanced glycation end products, ARB; angiotensin Ⅱ receptor blocker, DPP4i; dipeptidyl peptidase-4 inhibitors, HFmrEF; Heart Failure with mid-range Ejection Fraction, HFpEF; Heart Failure with preserved Ejection Fraction, HFrEF; Heart Failure with reduced Ejection Fraction, SGLT2i; sodium-glucose cotransporter 2 inhibitors. | | | | | | | | |
|  |  |  |  |  |  |  |  |  |
|  |  |  |  |  |  |  |  |  |
|  |  |  |  |  |  |  |  |  |
|  |  |  |  |  |  |  |  |  |
|  |  |  |  |  |  |  |  |  |

| Table S2. Univariate analysis of factors associated with MACCE | | | |
| --- | --- | --- | --- |
| Variable | HR [95% CI] |  | *P* Value |
| Cardiac rehabilitation, per times / month | 0.998 [0.809-1.230] |  | 0.998 |
| **Cardiovascular diseases** |  |  |  |
| HFrEF | 1.680 [0.735-3.841] |  | 0.219 |
| HFmrEF | 1.626 [0.574-4.608] |  | 0.361 |
| HFpEF | 1.560 [0.808-3.012] |  | 0.185 |
| Stable angina pectoris | 0.383 [0.168-0.875] |  | 0.023^*^ |
| Acute coronary syndrome | 0.717 [0.279-1.845] |  | 0.490 |
| **Medications** |  | | |
| ARB | 1.074 [0.556-2.073] |  | 0.832 |
| ACE | 1.369 [0.624-3.005] |  | 0.433 |
| β blocker | 1.704 [0.821-3.535] |  | 0.153 |
| Calcium blocker | 0.519 [0.227-1.186] |  | 0.120 |
| Diuretic | 3.150 [1.575-6.301] |  | 0.001^*^ |
| DPP4i | 0.468 [0.112-1.948] |  | 0.297 |
| Insulin | 0.049 [0.002-0.010] |  | 0.612 |
| Metformin | 0.330 [0.045-2.407] |  | 0.274 |
| SGLT2i | 0.801 [0.350-1.830] |  | 0.598 |
| ^*^; *P* < 0.050, ACE; angiotensin-converting enzyme inhibitor, AGEs; advanced glycation end products, ARB; angiotensin Ⅱ receptor blocker, CI; Confidence interval, DPP4i; dipeptidyl peptidase-4 inhibitors, HFmrEF; Heart Failure with mid-range Ejection Fraction, HFpEF; Heart Failure with preserved Ejection Fraction, HFrEF; Heart Failure with reduced Ejection Fraction, HR; Hazard Ratio, MACCE; major adverse cardiac and cerebrovascular events, SGLT2i; sodium-glucose cotransporter 2 inhibitors. | | | |

| Table S3. Multivariate analysis of factors associated with MACCE in physical functions | | | | | | |
| --- | --- | --- | --- | --- | --- | --- |
|  | HR [95% CI] | *P* Value | HR [95% CI] | *P* Value | HR [95% CI] | *P* Value |
| AGEs score | 1.038 [1.001-1.076] | 0.043^*^ | 1.037 [1.001-1.075] | 0.046^*^ | 1.015 [0.979-1.053] | 0.418 |
| Handgrip strength | 0.960 [0.923-0.998] | 0.039^*^ |  |  |  |  |
| Knee extension strength |  |  | 0.980 [0.956-1.005] | 0.122 |  |  |
| 6MWD |  |  |  |  | 0.995 [0.993-0.998] | 0.001^*^ |
| ^*^; *P* < 0.050, AGEs; advanced glycation end products, CI; Confidence interval, HR; Hazard Ratio, MACCE; major adverse cardiac and cerebrovascular events, 6MWD; 6-min walk distance. | | | | | | |
|  |  |  |  |  |  |  |
|  |  |  |  |  |  |  |

| Table S4. Multivariate analysis of factors associated with MACCE | | | | |
| --- | --- | --- | --- | --- |
|  | HR [95% CI] | *P* Value | HR [95% CI] | *P* Value |
| AGEs score | 1.043 [1.007-1.080] | 0.019^*^ | 1.039 [1.004-1.075] | 0.028^*^ |
| Stable angina pectoris | 0.386 [0.169-0.862] | 0.024^*^ |  |  |
| Diuretic |  |  | 3.035 [1.515-6.081] | 0.002^*^ |
| ^*^; *P* < 0.050, AGEs; advanced glycation end products, CI; Confidence interval, HR; Hazard Ratio, MACCE; major adverse cardiac and cerebrovascular events. | | | | |
|  |  |  |  |  |
|  |  |  |  |  |

Figure S1

Figure S2

Figure S3
